# Age Is Just A Number: Health Diagnoses as an Important Predictive Factor of Cognitive Functioning in Physicians

**DOI:** 10.1093/geroni/igaf122.4295

**Published:** 2025-12-31

**Authors:** Miranda McDaniel, Johanna Carrasco, Taylor Evans, Michael Williams, Betsy Williams

**Affiliations:** Professional Renewal Center, Lawrence, Kansas, United States; Professional Renewal Center, Lawrence, Kansas, United States; Professional Renewal Center, Lawrence, Kansas, United States; Professional Renewal Center/Wales Behavioral Assessment, Lawrence, Kansas, United States; Professional Renewal Center, Lawrence, Kansas, United States

## Abstract

The physician workforce is aging. The Federation of State Medical Boards 2024 census indicated that 31% of licensed physicians are 60 years and older1. Late career physicians are an invaluable community asset that comprise an increasing proportion of the physician workforce3. While it is true that aging is associated with changes in physical and cognitive functioning that can affect skills germane to clinical work, late career physicians have knowledge, skills, resilience, and wisdom equitable with, and in many cases greater than, younger colleagues4. To address any concerns that may arise as part of the normal aging process, some health systems are requiring late career physicians to complete a screening process once they reach a target age; for example, the Society of Surgical Chairs recommended mandatory cognitive and psychomotor testing beginning at age 653. Additional factors are associated with cognitive functioning such as medical health2. This study explores the contribution of several health diagnoses as predictive of overall cognitive functioning. This study utilizes a sample of physicians aged 23 - 84 years (N = 189, mean 56) referred for a comprehensive fitness for duty assessment. As part of the evaluation process, participants undergo a full neuropsychological battery including the Wechsler Adult Intelligence Scale (WAIS) 4th edition. Analyses indicate an association among age, physical health, and WAIS-4 Full Scale IQ. This research has implications for the implementation of additional screening measures for health and cognitive impairment across the health professional career span and reducing age related bias in the workplace.

